# Rational Use of Ceftriaxone in Necrotizing Fasciitis and Mortality Associated with Bloodstream Infection and Hemorrhagic Bullous Lesions

**DOI:** 10.3390/antibiotics11111454

**Published:** 2022-10-22

**Authors:** Hung-Yen Chen, Tsung-Yu Huang, Jiun-Liang Chen, Liang-Tseng Kuo, Kuo-Chin Huang, Yao-Hung Tsai

**Affiliations:** 1 Department of Orthopaedic Surgery, Chia-Yi Chang Gung Memorial Hospital, Taiwan 613, China; 2 Division of Infectious Diseases, Department of Internal Medicine, Chia-Yi Chang Gung Memorial Hospital, Taiwan 613, China; 3 College of Medicine, Chang Gung University at Taoyuan, Taiwan 333, China; 4 Microbiology Research and Treatment Center, Chia-Yi Chang Gung Memorial Hospital, Taiwan 613, China

**Keywords:** necrotizing fasciitis, bloodstream infection, hemorrhagic bullae, monomicrobial, Gram-negative, ceftriaxone

## Abstract

Necrotizing fasciitis (NF) is an uncommon life-threatening necrotizing skin and soft tissue infection. The formation of hemorrhagic bullae is a special skin sign of NF. The purposes of this study were to investigate the incidence of hemorrhagic bullae formation and bacteremia associated with different organisms, to appraise the appropriate use of ceftriaxone, and to compare the clinical and laboratory risk indicators of NF patients with Gram-positive and Gram-negative infections on the initial examination. **Methods**: From March 2018 to December 2020, there were seventy-four NF patients with positive monomicrobial bacterial cultures enrolled based on surgical confirmation, and were categorized into two groups: the Gram-positive group and the Gram-negative group. Ceftriaxone susceptibility tests were carried out using the standard disk diffusion technique. Data, such as demographics, clinical outcomes, microbiological results, presentations of hemorrhagic bullae, and laboratory findings, were compared among these two groups. **Results**: The Gram-negative group included 52 patients, of whom 6 patients died, resulting in a mortality rate of 11.5%. The Gram-positive group included 22 patients and none died. Patients with bacteremia, hemorrhagic bullae, shock, fever, higher segmented forms and banded forms, and lower platelet counts constituted higher proportions in the Gram-negative group than in the Gram-positive group. The multivariate analysis identified six variables for the differentiation of Gram-negative and Gram-positive NF: the presentation of bacteremia, hemorrhagic bullae, shock at first consultation, fever with body temperature > 38.5 °C, band forms > 0%, and segmented forms ≦ 74%. A total of 66 isolates (89.2%) was susceptible to ceftriaxone. **Conclusions**: Gram-negative NF patients were significantly associated with hemorrhagic bullae presentation, blood stream infection, and mortality. Physicians should be alert to NF patients with the appearance of bacteremia, shock, fever, higher WBC banded and segmented forms, and lower platelet counts at the emergency department, with patients revealed to more likely have Gram-negative infections. Ceftriaxone with/without other appropriate antibiotics under the supervision of infectious doctors appeared to be clinically effective for the treatment of NF and blood stream infections.

## 1. Introduction

Necrotizing fasciitis (NF) is a life-threatening skin and soft tissue infection, and even with a better understanding of the pathophysiology of this devastating infection, mortality remains high [1,2,3,4]. Patients with necrotizing fasciitis presenting with bacteremia, hemorrhagic bullae, subcutaneous bleeding, purpura, necrosis, and gangrene need to be diagnosed early at the time of presentation in the emergency room. Hemorrhagic bullae is a special skin sign that can be crucial for identifying and recognizing necrotizing fasciitis; moreover, it can even be the cause of devastating consequences [5,6,7,8,9]. The early recognition and diagnosis of NF using skin bullous lesions, purple skin discoloration, hypotension, and elevated laboratory markers can allow for the initiation of proper treatment strategies to decrease mortality rates [1,2,3,4,5,6,7,8,9,10,11,12,13].

*Vibrio* spp. was reported as the leading causative pathogen of NF and related fatalities in our institution, which is located in a warm-water coastal region in southwest Taiwan; therefore, we established a treatment strategy, including emergency fasciotomy or amputation, antibiotic therapy with a third-generation cephalosporin plus tetracycline, and admission to the intensive care unit (ICU) for patients with fulminant necrotizing fasciitis [12,13,14,15,16,17,18]. NF-associated bloodstream infections, especially of the *Vibrio* and *Aeromonas* species, are commonly found in our institution, and have been proven to increase mortality [15,17,18]. Although many studies have clearly demonstrated the mortality rate of different pathogens and treatment protocols, the knowledge surrounding the relationships between hemorrhagic bullae, bacteremia, and organisms is still lacking.

Vancomycin, linezolid, or clindamycin plus imipenem, meropenem, ampicillin/sulbactam, tigecycline and piperacillin/tazobactam, or ceftriaxone–metronidazole have been recommended for the initial antibiotic regimen for treating necrotizing fasciitis [19,20,21,22,23]. Our previous study advised the usage of ceftriaxone with/without other regimens at the first suspicion of necrotizing fasciitis to cover Gram-positive, Gram-negative, and anaerobic organisms [18]. However, ceftriaxone resistance has been increasing in the past decade, and the most reported strains have been the *Klebsiella* species, *Escherichia coli*, *Pseudomonas aeruginosa*, *Salmonella Typhi*, *Acinetobacter baumannii*, and *Staphylococcus aureus* [24,25,26]. Although these ceftriaxone-resistant pathogens might cause NF, the usage of ceftriaxone regarding the efficacy and safety in NF patients upon arrival to the emergency department remains questionable.

The purposes of this study were to investigate the incidence of hemorrhagic bullae formation and bacteremia associated with different organisms, to appraise the appropriate use of ceftriaxone, and to compare the clinical and laboratory risk indicators of NF patients with Gram-positive and Gram-negative infections on the initial examination.

## 2. Results

### 2.1. Microbiological Findings and Hemorrhagic Skin Presentation in NF Patients

According to the microbiological findings, the culture results from the wounds or blood specimens were classified into monomicrobial, polymicrobial, and no growth, in which 109 patients were included in this study. Eight patients died, resulting in a mortality rate of 7.3% (Table 1). Sixty patients presented hemorrhagic bullous formation, and forty-nine patients showed nonhemorrhagic skin lesions at the time of patient arrival at the emergency department or at the time of consultation in the hospital ward. All eight dead patients were revealed to have hemorrhagic bullous lesions. There were six patients with a polymicrobial infection and five of them were revealed to have hemorrhagic bullae.

**Figure 1:**
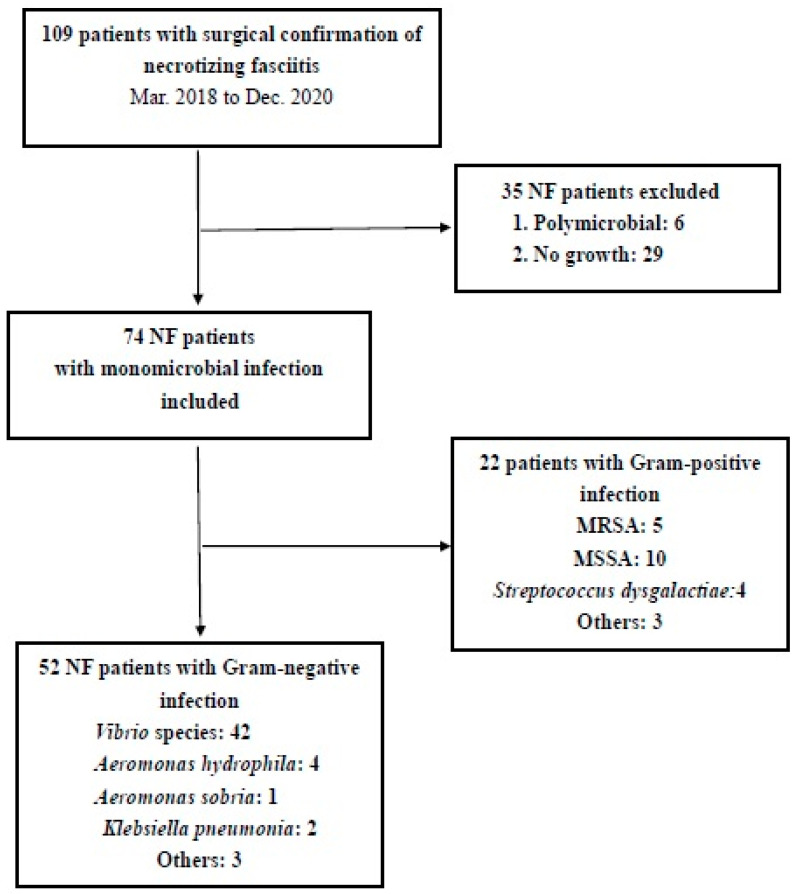
Flow chart of NF patient inclusion.

Seventy-four patients were found to have a monomicrobial infection (67.9%). Among the bacterial species identified, the *Vibrio* species was the most dominant pathogen (42 patients, 38.5%), followed by methicillin-sensitive *Staphylococcus aureus* (MSSA) (10 patients, 9.2%), methicillin-resistant *Staphylococcus aureus* (MRSA) (5 patients, 4.6%), *Aeromonas* spp. (5 patients, 4.6%), and *Streptococcus* *dysgalactiae* (4 patients, 3.7%). Six NF patients with a monomicrobial infection died, for which the pathogens responsible were *Vibrio vulnificus* (*n* = 3), *Vibrio cholerae* non-O1 (*n* = 1), *Aeromonas hydrophila* (*n* = 1), and *Klebsiella pneumonia* (*n* = 1) (Figure 1).

The pathogens identified in the polymicrobial cultures were Staphylococcus aureus, coagulase-negative Staphylococcus (CoNS), β-hemolytic Streptococcus, Escherichia coli, Enterobacter cloacae, Enterococcus faecalis, Parvimonas micra, Atropobium parvulum, Morganella morganii, and Klebsiella pneumonia. None of the patients with polymicrobial NF died. Bacterial growth was absent in 29 patients, of whom 5 presented with hemorrhagic bullae and 2 died.

Broad-spectrum antibiotics were initially administered to patients diagnosed with necrotizing fasciitis in the emergency room; ceftriaxone and doxycycline were prescribed in 33 cases, ceftriaxone alone in 67 cases, ceftriaxone and vancomycin in 6 case, piperacillin and vancomycin in 1 case, amoxicillin in 1 case, and clindamycin in 1 case. These antibiotics were continued after surgery and changed to antibiotics specially targeting cultured bacteria under the supervision of infectious doctors.

### 2.2. Patient Characteristics in the Gram-Negative Group

The mean age of the Gram-negative group was 69.7 years (range, 27 to 92 years). Six patients died a mean of 12.1 days after admission, resulting in a mortality rate of 11.5%. All patients initially underwent fasciotomy and debridement. One patient with a *Klebsiella pneumonia* infection had a below-the-knee amputation performed, and one patient with a *Vibrio vulnificus* infection underwent an above-the-knee amputation after a few days due to progressive skin involvement following fasciotomy. Fifteen patients received skin grafts and twenty-eight patients underwent a repeated debridement with wound care after initial fasciotomy. Seven patients did not undergo any surgery following fasciotomy, and three of them died.

Twenty-four patients had a history of hepatic dysfunction (liver cirrhosis, hepatitis B or C, hepatic cell carcinoma, or alcoholic liver disease) with/without other medical comorbidities (diabetes mellitus, chronic kidney disease, cancer, steroid usage, or gout). Ten patients had diabetes mellitus with/without other medical conditions. Five patients reported a history of heart disease, such as heart failure, hypertension, and coronary heart disease. Three patients had gout, two had steroid intake, two had chronic kidney disease, and one had colon cancer. Five patients did not have any chronic illnesses. Nineteen patients had a systolic blood pressure of ≤90 mmHg, and another nineteen patients had a body temperature of >38.5 °C.

**Figure 2:**
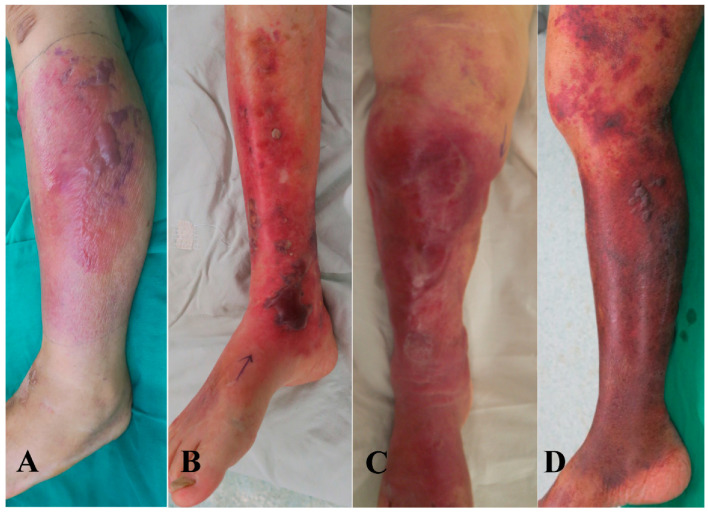
Skin lesions in patients with Gram-negative necrotizing fasciitis. (**A**) A 75 year-old patient with *Vibrio vulnificus* infection revealed to have hemorrhagic bullae lesions on left leg. (**B**) A 86 year-old male with *Aeromonas hydrophila* in wound culture of left leg revealed to have hemorrhagic bullae lesions. (**C**) A 81 year-old female with *Serratia marcescens* infection presented with lower right leg hemorrhagic bullae lesions. (**D**) A 78 year-old male with *Aeromonas sobria* infection in blood and wound cultures revealed to have lower right leg hemorrhagic bullae lesions.

Regarding positive cultures in the Gram-negative group, 13 were identified in wounds, 17 in blood, and 22 in both wounds and blood. The majority of Gram-negative pathogens was *Vibrio vulnificus*, which could easily cause hemorrhagic bullae and blood steam infections. There were 39 patients presenting bacteremia, of whom 5 died. Hemorrhagic bullous lesions were present in 43 patients, and 6 of them died (Figure 2).

### 2.3. Patient Characteristics in the Gram-Positive Group

The Gram-positive group had a mean age of 64.5 years (range of 47–84 years). All positive cultures were identified in wounds, and none of the patients died. All patients initially received a fasciotomy. Seven patients received skin grafts, and thirteen patients underwent a repeated debridement. Two patients received wound care without further surgical management.

**Figure 3:**
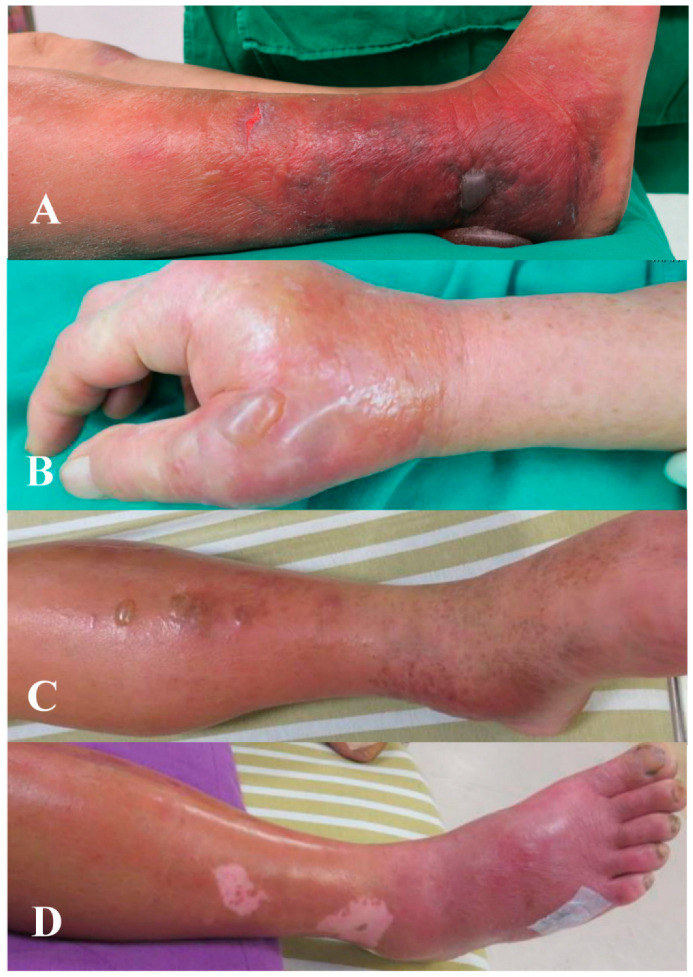
Skin lesions in patients with Gram-positive necrotizing fasciitis. (**A**) A 70 year-old female with MRSA infection revealed to have lower right leg hemorrhagic bullae lesions. (**B**) A 77 year-old female with *Streptococcus* *dysgalactiae* in wound culture of left hand revealed to have nonhemorrhagic bullae lesions. (**C**) A 54 year-old male with MSSA infection presented with nonhemorrhagic bullae on lower left leg. (**D**) A 59 year-old male with *Corynebacterium* infection revealed to have lower right leg nonhemorrhagic bullae lesions.

One patients had hepatitis C and diabetes mellitus. Six patients had a history of hepatic dysfunction with or without other comorbidities. Four patients had gout, three had diabetes mellitus, two had chronic kidney disease, and two had a steroid intake. One had seborrheic dermatitis, and one had parkinsonism. Two patients did not reported any underlying diseases. None of these patents were revealed to have a blood stream infection, and seven patients were observed to have hemorrhagic bullous lesions (Figure 3).

### 2.4. Antibiotic Susceptibility and Resistance of Gram-Negative and Gram-Positive Pathogens

Isolates of *Vibrio vulnificus*, *Vibrio parahemolytics*, *Klebsiella pneumonia*, *Escherichia coli*, and *Serratia* *marcescens* were susceptible to amikacin, ceftazidime, ceftriaxone, ertapenem, levofloxacin, and tetracycline. The *Vibrio cholerae* non-O1 isolate was susceptible to ceftriaxone, but resistant to ampicillin, chloramphenicol, sulfamethoxazole–trimethoprim, and tetracycline. The isolates of five *Aeromonas* patients were susceptible to amikacin (AMK), ceftazidime, ceftriaxone, cefuroxime, ciprofloxacin, ertapenem, and gentamicin. Three *Aeromonas* isolates were susceptible to ertapenem and tetracycline; however, two isolates were resistant to them. An *Achromobacter xylosoxidans* isolate was susceptible to ceftazidime, ciprofloxacin, colistin, imipenem, and levofloxacin, with resistance to amikacin, ciprofloxacin, cefepime, and gentamicin.

Except for MRSA, *Corynebacterium falsenii*, and *Bacillus*, 15 isolates of Gram-positive pathogens were susceptible to oxacillin, ampicillin, clindamycin, teicoplanin, vancomycin, and ceftriaxone. All MSSA isolates were resistant to erythromycin and penicillin, two *Streptococcus* *dysgalactiae* isolates were resistant to clindamycin and erythromycin, and one *Staphylococcus epidermidis* isolate was resistant to oxacillin and penicillin.

A total of 66 isolates (89.2%) was susceptible to ceftriaxone with either Gram-positive or Gram-negative pathogens (Table 2).

### 2.5. Comparison of Gram-Negative and Gram-Positive Groups

The patients with Gram-negative NF had a significantly higher incidence of bacteremia, shock, fever, higher banded and segmented forms of white blood cell (WBC) counts, and lower platelet counts than patients with Gram-positive NF infections in the emergency room (Table 2). Gram-negative pathogens caused significantly higher presentations of bacteremia and hemorrhagic bullae, which were associated with death (Table 3). The multivariate analysis identified six variables for differentiating Gram-negative and Gram-positive NF: the presentation of bacteremia, hemorrhagic bullae, shock at first consultation, fever with a body temperature of > 38.5 °C, band forms of WBC > 0%, and segmented forms of WBC ≦ 74% (Table 4).

## 3. Discussion

Monomicrobial necrotizing fasciitis was reported to have increased in incidence in the recent decade, especially Gram-negative NF, reported to have more progressive clinical courses and a higher mortality than Gram-positive NF [15,18,27,28,29,30]. Cutaneous features of NF were divided into three stages, and hemorrhagic bullae appeared in the third stage [5,6]. Wang et al. reported that 68% patients developed hemorrhagic bullae on the fourth day; however, our patients commonly presented with hemorrhagic bullous skin lesions upon arrival to the ER [5]. Thus, the appearance of hemorrhagic bullae was an important diagnostic criteria for NF in our institution, especially in those patients with *Vibrio* and *Aeromonas* infections [7,8,9,12,13,14,15,16,17,18]. Many of the literature had proved that Gram-negative NF had a significant higher prevalence of bloodstream infections than Gram-positive NF [9,13,14,15,28,29,30,31]. In this study, we demonstrated that Gram-negative NF with hemorrhagic bullae presentation and bacteremia was significantly associated with poor prognosis, which should be considered first when patients are revealed to have quickly progressing and fulminant features at the ER.

Besides emergent surgical interventions, appropriate broad-spectrum antibiotics were also an important strategy for NF patients at first onset. Ceftriaxone is one of the commonly utilized antibiotics due to its commendable antibacterial efficacy and excellent activity against many Gram-negative and most Gram-positive bacteria in patients with bacteremia, pneumonia, a urinary tract infection, necrotizing soft tissue infection, bone and joint infections, and bacterial meningitis [29,30,31,32,33]. Although the increasing prevalence of ceftriaxone-resistant Gram-negative pathogens has been reported as a concerning issue in critically ill patients, ceftriaxone is still recommended to be effective for blood stream infections and various community-acquired infections due to its excellent safety profile in many literatures [24,25,26,34,35,36,37]. Our study demonstrated that 89.2% of Gram-positive and Gram-negative pathogens was susceptible to ceftriaxone. We also observed that Gram-negative NF patients had a higher incidence of bloodstream infections (39/52), and nearly all Gram-negative pathogens (51/52) were susceptible to ceftriaxone. With the emergence of surgical interventions and appropriate antibiotics under the supervision of infectious doctors, the mortality rate of 15.4% during the 4-year period from 2007 to 2010 was reduced to a mortality rate of 7.3% during a 3-year period from 2018 to 2020. The rational use of ceftriaxone with/without other antibiotics as an empirical therapy in necrotizing fasciitis and bloodstream infections could be verified to have sufficient clinical efficacy.

NF requires teams to work towards acting swiftly on early suspicions, conducting immediate surgical interventions, and providing aggressive care, which can successfully decrease mortality; therefore, clinical presentations and laboratory risk indicators are more important for the rapid recognition and management of NF [7,8,9,12,13,14,15,16,17,18]. In this prospective study, we confirmed that NF patients with any two or more indicators, including a presentation of bacteremia, hemorrhagic bullous lesions, shock at first consultation, fever with a body temperature of > 38.5 °C, band forms of > 0%, and segmented forms of ≦ 74%, at the time of the initial consultation in the ER or ward should be assumed to have the Gram-negative infection first, due to being capable of causing more devastating outcomes.

A strength of this study was that we prospectively took pictures of every NF patient’s lesions on arrival to the ER, and were defined as hemorrhagic or nonhemorrhagic skin lesions by two doctors (Y.H.T. and T.Y.H.) to increase the reliability of the assessment.

There were several limitations to our study. First, the number of enrolled patients was still too small, and a further multi-institutional cohort study may be needed. Second, 26.7% of our NF patients had no bacterial growth found in blood or wound specimens. Many molecular methods, such as the simple polymerase chain reaction (PCR), multiplex PCR, real-time PCR, nucleic acid sequence-based amplification (NASBA), loop-mediated isothermal amplification (LAMP), and oligonucleotide DNA microarray, can be considered for the detection of diverse pathogens.

## 4. Materials and Methods

### 4.1. Patient Selection

We conducted a prospective cohort study and evaluated those patients who were initially diagnosed with NF of the limbs by emergency medicine doctors, and who had an excisional fasciotomy or an immediate limb amputation performed by orthopedic surgeons between March 2018 and December 2020 in Chia-Yi Chang Gung Memorial Hospital. Routine blood cultures were collected at the ER, and wound specimens were obtained with sterile cultrate swabs during surgery. The cultured specimens of patients were confirmed with a microbiologic evaluation a few days after surgery, and were classified as a monomicrobial infection, polymicrobial infection, and no growth. We excluded those patients with prosthetic joint infections, surgical complicated wounds, osteomyelitis, and pre-existing chronic ulcers.

### 4.2. Diagnosis and Treatment Protocol

NF was defined by the surgically findings of the presence of grayish necrotic soft tissue and hemorrhagic bullae, the loss of resistance in normally adherent fascia to digital blunt dissection, and the appearance of pus with a foul dishwater odor. Broad-spectrum antibiotics were initially administered to all patients, and an emergent fasciotomy or immediate limb amputation was immediately performed in other cases when necrotizing fasciitis was diagnosed.

### 4.3. Clinical Assessment

Demographic data, including the presentation of hemorrhagic bullae, nonhemorrhagic serous-filled bullae, and mortality, were reviewed for each patient according to microbiological findings. To assess clinical outcomes after treatment, mortality was defined as death due to progressive sepsis or medical complications within 6 months after the first surgery.

Further, we enrolled NF patients with monomicrobial infections and categorized them into two groups: the Gram-positive group and the Gram-negative group. Differences in mortality, clinical outcomes, laboratory findings, antibiotic resistance, the presentation of bacteremia, and the presentation of hemorrhagic bullae were compared between the Gram-positive and Gram-negative groups.

### 4.4. Microbiology Laboratory Procedures

Isolates of pathogens from primary cultures were identified by their colonial appearance, Gram stain, agglutination with specific antisera, and with conventional biochemical tests used in clinical microbiology laboratories. Matrix-assisted laser desorption/ionization-time of flight mass spectrometry (MALDI-TOF MS) was used to identify those isolates to a species level.

The antimicrobial susceptibility of pathogens was analyzed in the hospital’s microbiology laboratory via the standard disk diffusion technique. These antimicrobial susceptibility tests were performed as recommended by the Clinical and Laboratory Standards Institute (CLSI), and the results were interpreted according to the CLSI criteria for these microorganisms. When the antimicrobial susceptibility test of the isolate revealed resistance in more than one antibiotic, the isolate of the patient was recorded to be an antibiotic-resistant case.

### 4.5. Statistical Analysis

Statistical analyses were performed with the use of Statistical Product and Service Solutions (SPSS) Version 18.0 statistical software (SPSS, Chicago, IL, USA). We used the two-tailed *t*-test for continuous variables and the Fisher exact test for categorical variables to examine the significant relationships between risk factors and outcomes between the Gram-positive and Gram-negative groups. To identify the diagnostic risk indicators, multivariate logistic regression was used to estimate the relative effect of variables by calculating unadjusted odds ratios for categorical outcomes. A value of *p* < 0.05 (two tailed) was considered significant.

## 5. Conclusions

NF patients accompanied by hemorrhagic bullae presentations and blood stream infections appeared to were associated with Gram-negative bacteria infections and mortality. Physicians should be alert to NF patients with the appearance of bacteremia, shock, fever, higher WBC banded and segmented forms, and lower platelet counts at the emergency department, which were revealed to more likely have the Gram-negative infection. Ceftriaxone with/without other appropriate antibiotics under the supervision of infectious doctors appeared to have a clinical effectiveness for the treatment of NF and bloodstream infection.

## Figures and Tables

**Table 1:** Mortality and microbiology in patients with necrotizing fasciitis.

	No. of	Hemorrhagic	Nonhemorrhagic	Bacteremia	Mortality
Variable	Patients	Bullae	Skin Lesions		
**Monomicrobial Infection**	**74**	**50**	**24**	**39**	**6 (8.1%)**
Gram-positive pathogens	**22**	**7**	**15**	**0**	**0 (0%)**
MRSA	5	4	1	0	0
MSSA	10	2	8	0	0
*Streptococcus* *dysgalactiae*	4	1	3	0	0
*Corynebacterium falsenii*	1	0	1	0	0
*Staphylococcus epidermidis*	1	0	1	0	0
*Bacillus*	1	0	1	0	0
Gram-negative pathogens	**52**	**43**	**9**	**39**	**6 (11.5%)**
*Vibrio vulnificus*	40	32	8	31	3
*Vibrio cholerae* non-O1	1	1	0	0	1
*Vibrio parahemolytics*	1	1	0	1	0
*Aeromonas hydrophila*	4	4	0	1	1
*Aeromonas sobria*	1	1	0	1	0
*Klebsiella pneumonia*	2	1	1	2	1
*Serratia marcescens*	1	1	0	1	0
*Escherichia coli*	1	1	0	1	0
*Achromobacter xylosoxidans*	1	1	0	1	0
**Polymicrobial Infection**	**6**	**5**	**1**	**1**	**0 (0%)**
**No growth**	**29**	**5**	**24**	**0**	**2 (6.9%)**
**Total**	**109**	**60**	**49**	**40**	**8 (7.3%)**

**Table 2:** Susceptible and resistant antibiotics in monomicrobial necrotizing fasciitis.

	No. of	Ceftriaxone-	Ceftriaxone-	Other Antibiotic-
Variable	Patients	Susceptible	Resistant	Resistant
Gram-positive pathogens	**22**	**16**	**6**	
MRSA	5	0	5	OXA, AMP, ERY, CLI
MSSA	10	10	0	ERY, PEN
*Streptococcus dysgalactiae*	4	4	0	CLI, ERY
*Corynebacterium falsenii*	1	0	1	CAP, ERY, CIP
*Staphylococcus epidermidis*	1	1	0	OXA, PEN
*Bacillus*	1	0	1	PEN, TRC, ERY, AMP
Gram-negative pathogens	**52**	**51**	**1**	
*Vibrio vulnificus*	40	40	0	None
*Vibrio cholerae* non-O1	1	1	0	AMP, CAP, SMX–TMP, TRC
*Vibrio parahemolytics*	1	1	0	CFU, CEZ, CIP
*Aeromonas hydrophila*	4	4	0	ERT, TRC
*Aeromonas sobria*	1	1	0	ERT, TRC
*Klebsiella pneumonia*	2	2	0	None
*Serratia marcescens*	1	1	0	CFU, CEZ
*Escherichia coli*	1	1	0	None
*Achromobacter xylosoxidans*	1	0	1	AMK, CIP, CEF, GM
**Total**	**74**	**66**	**6**	

OXA, oxacillin; ERY, erythromycin; AMP, ampicillin; CLI, clindamycin; PEN, penicillin; CAP, chloramphenicol; SMX-MP, sulfamethoxazole–trimethoprim; TRC, tetracycline; CFU, cefuroxime; CEZ, cefazolin; CIP, ciprofloxacin; ERT, ertapenem; AMK, amikacin; CEF, cefepime; GM, gentamicin.

**Table 3:** Comparison between the Gram-negative NF group and Gram-positive NF group for characteristics and laboratory data at first consultation in the ER.

		Gram-Negative Group (N = 52)	Gram-Positive Group (N = 22)	*p* Value
Age (years)		69.7	64.5	
Sex				
Male		38	15	
Female		14	7	
Mortality rate (%)		11.5	0	
Deaths		6	0	
Survivals		46	22	
Positive culture				
Wound		13	22	
Blood		17	0	
Wound and blood		22	0	
Isolates of ceftriaxone susceptible		51	15	
Presentation of bacteremia		39	0	0.0001 *
Death	5		0	
Presentation of hemorrhagic bullae ^a^		43	7	0.0001 *
Death	6		0	
Survivals	37		7	
Systolic blood pressure (mm Hg) ^a^				0.0004 *
≦90		19	0	
>90		33	22	
Body temperature (°C) ^a^				0.023 *
>38.5		19	2	
<38.5		33	20	
White blood cell	Mean	13,528.8 ± 8497.7	16,195.4 ± 6614.1	0.19
counts (cells/mm^3^) ^b^				
Band forms (%) ^b^	Mean	8.3 ± 9.8	3.6 ± 6.4	0.04 *
0		9	9	0.0406 *^a^
>0		43	13	
Segmented forms (%) ^b^	Mean	76.8 ± 14.7	84.3 ± 7.9	0.026 *
≦74		17	2	0.0425 *^a^
>74		35	20	
Lymphocyte forms (%) ^b^	Mean	8.1 ± 6.0	6.3 ± 4.4	0.19
Platelet counts (per mm^3^) ^b^	Mean	134,815 ± 72,698	247,636 ± 154,001	0.0001 *
≦150,000		26	6	0.08 ^a^
>150,000		26	16	
Albumin (g/dL) ^b^	Mean	3.49 ± 0.68	3.71 ± 0.49	0.16

* mean *p* < 0.05 and the difference was significant; values were expressed as number of patients and median (SD); ^a^ Fisher’s exact test; ^b^ *t*-test.

**Table 4:** Diagnostic risk factors for differentiation of Gram-negative and Gram-positive monomicrobial necrotizing fasciitis identified with multivariate analysis.

	Odds Ratio	*p* Value ^a^
Variable	(95% Confidence Interval) ^†^	
Bacteremia	131.667 (7.461–2321.745)	0.0009 *
Hemorrhagic bullae	10.238 (3.244–32.314.925)	0.0001 *
Systolic blood pressure ≦ 90 mmHg	26.194 (1.504–456.271)	0.0251 *
Body temperature > 38.5	5.758 (1.211–27.381)	0.0278 *
Band forms > 0%	0.302 (0.099–0.920)	0.0351 *
Segmented forms ≦ 74%	4.857 (1.016–23.226)	0.0477 *

* mean *p* < 0.05 and the difference was significant; ^†^ adjusted ORs and 95% CIs were estimated using multivariate logistic regression. ^a^ Fisher’s exact test.

## Data Availability

The datasets used and/or analyzed during the current study are available from the author (orma2244@adm.cgmh.org.tw) on reasonable request.

## Abbreviations

NF: necrotizing fasciitis; ER, emergency room; CLSI, Clinical and Laboratory Standards Institute; *S. aureus*, *Staphylococcus aureus*; *V. vulnificus*, *Vibrio vulnificus*; MRSA, methicillin-resistant *Staphylococcus aureus*; MSSA, methicillin-sensitive *Staphylococcus aureus*; ICU, intensive care unit; PCR, polymerase chain reaction; WBC, white blood cell.
